# Fibroblasts promote the collective invasion of ameloblastoma tumor cells in a 3D coculture model

**DOI:** 10.1002/2211-5463.12313

**Published:** 2017-11-02

**Authors:** Takao Fuchigami, Hirofumi Koyama, Michiko Kishida, Yoshiaki Nishizawa, Mikio Iijima, Toshiro Kibe, Masahiro Ueda, Tohru Kiyono, Yoshimasa Maniwa, Norifumi Nakamura, Shosei Kishida

**Affiliations:** ^1^ Department of Oral and Maxillofacial Surgery Kagoshima University Graduate School of Medical and Dental Sciences Japan; ^2^ Department of Biochemistry and Genetics Kagoshima University Graduate School of Medical and Dental Sciences Japan; ^3^ Division of Carcinogenesis and Cancer Prevention National Cancer Center Research Institute Tokyo Japan; ^4^ Division of Thoracic Surgery Kobe University Graduate School of Medicine Hyogo Japan

**Keywords:** 3D culture, ameloblastoma, collective cellular invasion, odontogenic tumor, stromal fibroblast

## Abstract

Ameloblastoma is a benign tumor of the odontogenic epithelium with several histological subtypes. All subtypes of ameloblastoma contain abundant stroma; the tumor cells invade collectively into the surrounding tissues without losing intratumor cell attachments. However, the molecular mechanisms mediating ameloblastoma invasion remain unclear. Here, we evaluated the functional significance of the interactions between ameloblastoma tumor cells and stromal fibroblasts on collective cellular invasion using a three‐dimensional cultivation method, double‐layered collagen gel hemisphere (DL‐CGH) culture. The AM‐1 plexiform and AM‐3 follicular human ameloblastoma cell lines and HFF‐2 human fibroblasts were labeled with GFP and DsRed, respectively. Collective cellular invasion of ameloblastoma cells was assessed in the presence or absence of fibroblasts. Notably, without fibroblasts, AM‐1 cells formed sharp, plexiform‐like invasive processes, whereas AM‐3 cells formed a series of blunt processes often observed during collective migration. In comparison, under the cocultures with HFF‐2 fibroblasts, AM‐3 cells formed tuft‐like invasive processes and collectively invaded into outer layer more than that observed with AM‐1 cells. Moreover, HFF‐2 fibroblasts localized to the tips of the invasive tumor processes. These findings suggest that tumor‐associated cells assist tumor cell invasion. Microscopic analysis of sectioned three‐dimensional cultures revealed that AM‐3/HFF‐2 hemispheres were histologically similar to follicular ameloblastoma tumor samples. Therefore, our findings suggest that ameloblastoma subtypes exhibit distinct invasion patterns and that fibroblasts promote collective tumor invasion in follicular ameloblastoma.

**Abbreviations**
3D: three‐dimensional
DL‐CGH: double‐layered collagen gel hemisphere
IL: interleukin
RT‐qPCR: quantitative real‐time reverse transcription polymerase chain reaction

Ameloblastoma is a benign odontogenic tumor with a high rate of recurrence after surgical resection compared with another odontogenic tumor types; therefore, treatment often includes a wide resection of the jaw 1, 2, 3, 4. As such, postoperative facial deformities and difficulties with eating and speaking can become major problems for patients 5. It was suggested that ameloblastoma cells induced osteoclastogenesis 6, and matrix metalloproteinase (MMP) secretions from tumor cells and surrounding osteoclast activation may facilitate this process 7, 8. However, the details remain unclear.

Ameloblastoma is classified into several histologic types, each with its own invasive growth pattern 1. The most common subtype of this disease is the follicular type, which is similar to the epithelial component of the enamel organ within a fibrous stroma. The ameloblasto‐like cuboidal tumor cells form a palisading pattern with reverse polarity. The second most popular subtype is the plexiform type, which is composed of anastomosing strands of ameloblastomatous epithelium 9. Stem cell markers of CD90 were reported to express differentially depending upon pathological types 10.

All tumors contain abundant tumor stroma including mesenchymal stromal cells surrounding the parenchyma. Moreover, pathological images of all types of this tumor demonstrate that ameloblastoma cells invade collectively into surrounding tissues without disrupting their cell–cell contacts 4, 11, 12. Collective cellular invasion is considered to be important during morphogenesis and in pathological processes such as wound healing and cancer cell invasion 13, 14, 15, but the details of this invasive mechanism are unknown. To the best of our knowledge, no reports have examined cellular factors that may affect the different styles of invasive growth in ameloblastoma. In addition, difficulties in culturing primary ameloblastoma cells and establishing immortalized derivatives have also hindered molecular analyses in these tumors 16. Ameloblastoma‐associated stroma is also considered important for the understanding of its pathological role 17, 18.

In this study, we used immortalized AM‐1 plexiform and AM‐3 follicular ameloblastoma cell lines to compare the differences between these two tumor types 16, 19. Our group previously found that interactions between tumor cells and stromal fibroblasts promote ameloblastoma cell migration and proliferation 20. Recently, stromal cells have been shown to play key roles in the pathophysiology of many diseases. For example, fibroblasts surrounding malignant tumors facilitate tumor cell growth and invasion 21. Similarly, cytokines and growth factors secreted by interstitial cells were also sufficient to alter tumor cell behavior 22. In ameloblastoma, several factors were reported to be included in the tumor–stroma interactions. For example, ameloblastoma‐derived CCN2 and TGF‐β regulated fibrogenesis and osteoclastogenesis, respectively 23.

Other group reported that the gene mutations such as SMO and BRAF existed in ameloblastoma and that the mutation pattern differed between plexiform type and follicular type 24. We have shown that different types of ameloblastoma cells have characteristic properties in gene expression pattern and invasion activity 19. Based on these findings, we hypothesized that intercellular interactions between ameloblastoma cells and the neighboring stroma could also affect the collective invasion of ameloblastoma cells. To analyze collective invasion in a physiologically relevant environment, we utilized a double‐layered collagen gel hemisphere (DL‐CGH)‐three‐dimensional (3D) culture method.

While several studies have focused on ameloblastoma tumor classification, little is known on their molecular pathophysiology, which has substantially hindered clinical decision making. Thus, clarification of these biological mechanisms will provide a foundation to develop new treatment strategies for this disease.

## Materials and methods

### Reagents

Dulbecco's modified Eagle's medium (DMEM) and Ham's F‐12 media were purchased from Nissui Corp. (Tokyo, Japan). Y‐27632 was purchased from Ado Q Bioscience (Irvine, CA, USA). Hydrocortisone and insulin were purchased from Wako Pure Chemical (Osaka, Japan). Recombinant human epidermal growth factor (EGF) was purchased from Invitrogen Corp. (Carlsbad, CA, USA).

### Vector construction and lentiviral transduction of fluorescence genes into ameloblastoma cell lines and fibroblast cell line

The construction and transduction of GFP‐ and DsRed‐expressing lentiviral vectors into ameloblastoma tumor cells (AM‐1, AM‐3) and HFF‐2 fibroblasts were performed as described previously 19. Briefly, cDNA‐encoding the fluorescent tags were inserted into the CSII‐CMV‐RfA lentiviral vector (provided by Miyoshi, RIKEN BioResource Center, Tokyo, Japan) with LR Gateway reactions (Invitrogen Corp.). The production of recombinant lentivirus with the vesicular stomatitis virus G glycoprotein has been described previously 25. Virus titers were estimated by quantitative real‐time reverse transcription polymerase chain reaction (RT‐qPCR) and an appropriate volume of viral supernatant was added to ameloblastoma (AM‐1 and AM‐3) and HFF‐2 cell cultures in the presence of G418 (400 μg·mL^−1^) and puromycin (10 μg·mL^−1^), to generate GFP‐expressing ameloblastoma cells and DsRed‐expressing HFF‐2 fibroblasts, respectively. After transduction, cells displaying continuous proliferation were selected for further analysis.

### Cell culture

AM‐1 and AM‐3 cells were established from human plexiform and follicular ameloblastoma, respectively 16, 19. GFP‐labeled AM‐1 and AM‐3 ameloblastoma cells were maintained with F‐medium (DMEM/Ham's F‐12 = 1 : 3) containing 5% FBS, insulin (10 μg·mL^−1^), Y‐27632 (20 μm), recombinant human EGF (0.2 μg·mL^−1^), adenine‐HCl (0.3 mg·mL^−1^), and hydrocortisone (2 μg·mL^−1^). DsRed‐labeled HFF‐2 fibroblasts were maintained with DMEM containing 10% FBS.

### Double‐layered collagen gel hemisphere culture

Double‐layered collagen gel hemisphere culture method was performed as previously described 26. Briefly, acid‐soluble collagen I (Nitta Gelatin Inc., Osaka, Japan), 10× Ham's F‐12 medium, and reconstruction buffer (2.2 g NaHCO_3_ and 4.77 g HEPES in 100 mL of 0.05 N NaOH) were mixed at a volume ratio of 8 : 1 : 1 and then used to seed cultured cells at a density of 3.0 × 10^6^ cells·mL^−1^. Alternatively, 5 μL of the mixture containing 3.0 × 10^4^ tumor cells or 1.5 × 10^4^ each of tumor cells and fibroblasts was dropped into wells in 12‐well plates. Once the mixture had gelled, a second 40 μL drop of collagen with 3.0 × 10^4^ HFF‐2 fibroblasts was placed directly on the top of the first drop, encapsulating it completely. The gel hemisphere was then submerged in the medium and cultured.

**Figure 1:**
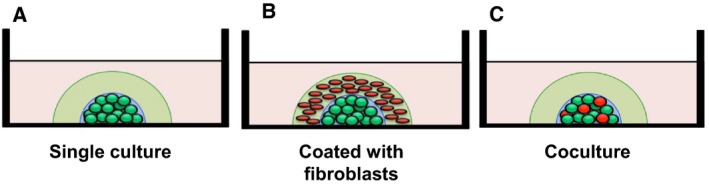
Experimental schematic representation of DL‐CGH culture. Green and red circles indicate GFP‐labeled ameloblastoma and DsRed‐labeled HFF‐2 fibroblast cells, respectively. (A) Single culture of ameloblastoma cells. (B) Ameloblastoma cells coated with the HFF‐2 fibroblast‐containing collagen gel. (C) Ameloblastoma cells and fibroblasts were mixed and cocultured in the inner layer.

We tried to simulate the intercellular interaction between ameloblastoma cells and fibroblasts. We adopted *in vitro* DL‐CGH 3D cultures and tested three ways of culture, namely single culture, coated culture, and coculture with ameloblastoma cells and fibroblasts (Fig. 1).

### Microscopy

Microscopic images were obtained with an ECLIPSE Ti‐E microscope (Nikon Corp., Tokyo, Japan) equipped with a PowerShot A640 camera (Canon Inc., Tokyo, Japan) as described previously 19. Fluorescent images of DL‐CGH culture were obtained using a conventional epifluorescent microscope (BZ‐X700; KEYENCE, Osaka, Japan).

## Results

### Histology

**Figure 2:**
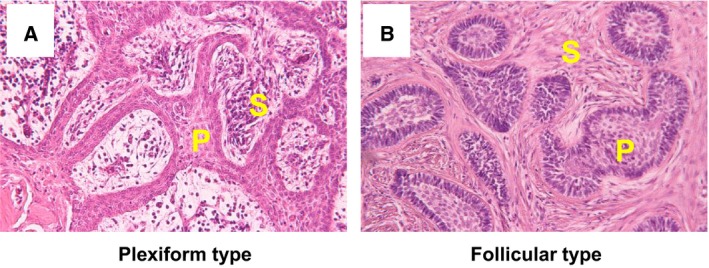
The pathologic images of plexiform (A) and follicular (B) ameloblastoma. (hematoxylin and eosin stain) P, tumor parenchyma; S, tumor stroma.

Typical histopathological findings are shown in Fig. 2. The plexiform type shows characteristics such as inconspicuous stellate reticulum and cyst‐like stromal degeneration (Fig. 2A). The follicular type has outer palisaded ameloblast‐like cells with inner zonal triangular‐shaped cells (Fig. 2B).

### Live cell imaging of ameloblastoma cells and fibroblasts

**Figure 3:**
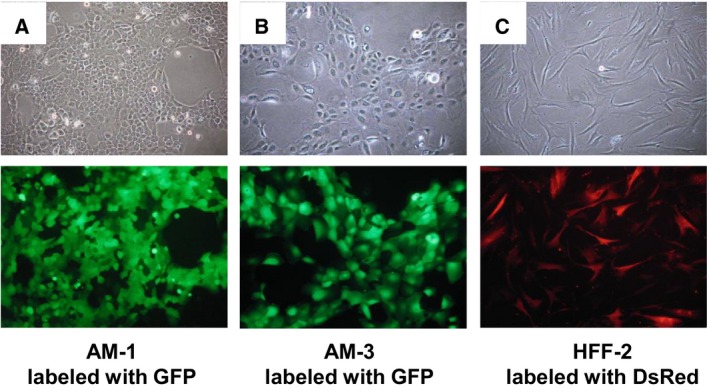
The images of ameloblastoma and fibroblast cell lines. AM‐1 plexiform (A) and AM‐3 follicular (B) ameloblastoma cells were labeled with GFP. (C) HFF‐2 fibroblasts were labeled with DsRed. Upper panels: phase‐contrast images. Lower panels: fluorescent images.

Ameloblastoma cells were labeled with GFP (Fig. 3A,B) and fibroblasts were labeled with DsRed, respectively (Fig. 3C). These labeling methods with fluorescent proteins enable us to visualize clearly ameloblastoma cells and fibroblasts without immunological staining.

### Single culture of ameloblastoma in DL‐CGH system

**Figure 4:**
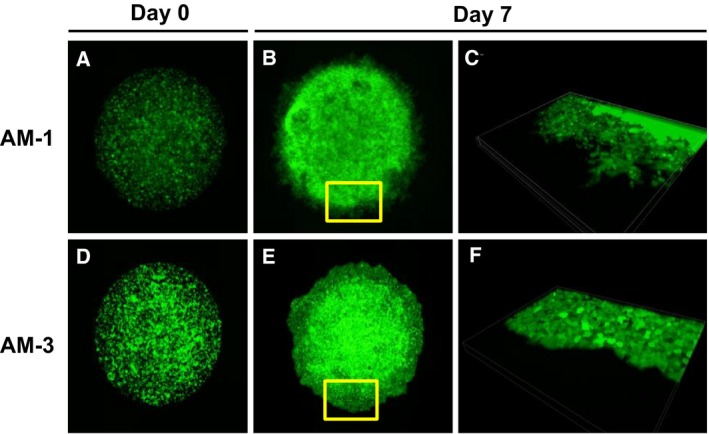
Microscopic images of ameloblastoma in single cultures using DL‐CGH. (A–C) AM‐1 cells. (D–F) AM‐3 cells. Day 0 (A,D) and Day 7 (B,C,E,F). Panels of C and F indicate 3D images of the area which are yellow‐boxed in B and E, respectively.

Double‐layered collagen gel hemisphere cultures with only AM‐1 or AM‐3 cells revealed that tumor cells invaded collectively remaining intercellular attachment and clearly demonstrated the differences in collective invasive potential between AM‐1 cells and AM‐3 cells. Specifically, plexiform AM‐1 cells formed small and sharp invasive processes (Fig. 4A–C), whereas follicular AM‐3 cells formed a series of blunt processes (Fig. 4D–F).

### DL‐CGH cultures of ameloblastoma cells coated with fibroblast‐containing collagen gel

**Figure 5:**
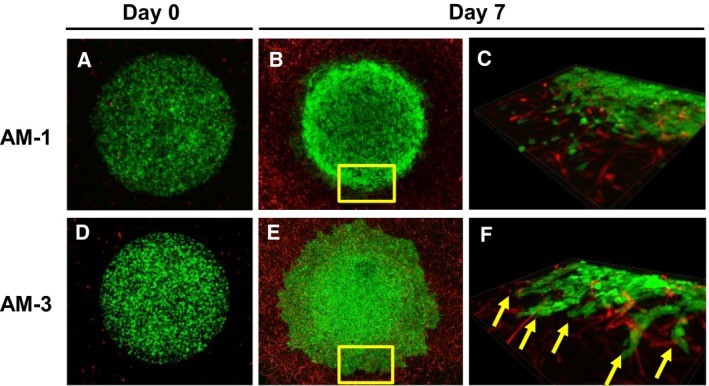
Microscopic images of DL‐CGH culture ameloblastoma cells coated with HFF‐2 fibroblast‐containing gel. (A–C) AM‐1 cells. (D–F) AM‐3 cells. Day 0 (A,D) and Day 7 (B,C,E,F). Panels of C and F indicate the magnified 3D images which are yellow‐boxed in panels B and E, respectively. Arrows indicate the tuft‐like blunt processes and tip‐associated fibroblasts.

The use of GFP‐labeled ameloblastoma‐derived cells coated with DsRed‐labeled HFF‐2 fibroblasts enabled the clear identification of collective tumor cell invasion in 3D cultures (Fig. 5). Both tumor subtypes displayed more abundant processes and enhanced invasive potential, in the presence of fibroblasts. AM‐3 cells formed more tuft‐like large processes than that observed without fibroblasts (Figs 4F and 5F). In addition, the fibroblasts localized to the tips of several tumor processes and seemed to potentiate invasion (Fig. 5F).

### Ameloblastoma and fibroblast DL‐CGH cocultures

**Figure 6:**
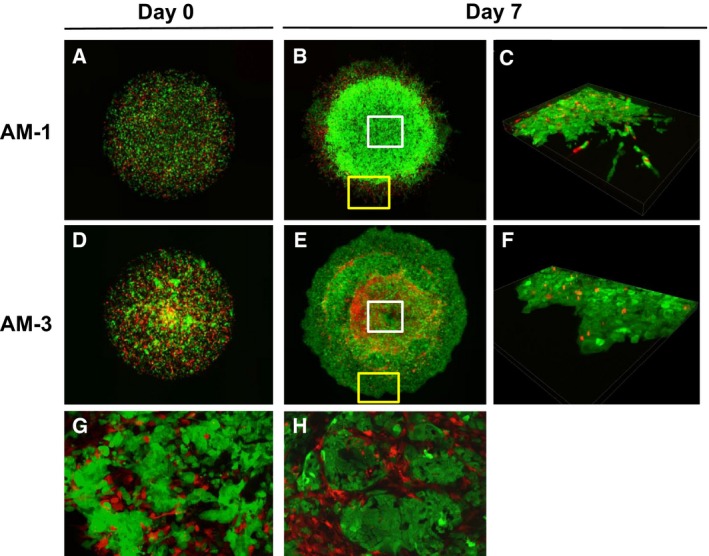
Ameloblastoma/fibroblast cocultures using DL‐CGH culture. (A–C,G) AM‐1 cells. (D–F,H) AM‐3 cells. Day 0 (A,D) and Day 7 (B,C,E–H). Panels of C and F indicate 3D images of the area in the yellow boxes shown in B and E, respectively. Panels G and H indicate full‐focused magnified images of the area in the white boxes shown in panels B and E, respectively.

We then evaluated collective cellular migration in ameloblastoma cells cocultured with fibroblasts by using the DL‐CGH culture system (Fig. 6). Interestingly, invasive processes were not as pronounced in tumor cells as compared to single cultures (Fig. 6C,F). However, microscopic imaging revealed that the centers of AM‐3/fibroblast hemispheres were histologically similar to follicular ameloblastoma tumors (Figs 2B and 6H), wherein fibroblasts surrounded the tumor cell colony to preserve intratumor cell adhesion.

## Discussion

Ameloblastoma is an odontogenic tumor that can be classified into several types with distinct invasive growth patterns based on histopathological findings 1. However, the molecular mechanisms governing tumor invasion remain unclear. As such, these tumors are often treated empirically by mandibular dissection and/or wide surgical resection 4. Several studies have evaluated gene expression, migration, invasive ability, and other characteristics in ameloblastoma cells. In addition, previous reports suggested that the variable expression of MMPs and cytokines may be responsible for the observable differences in tumor histology between subtypes 27, 28, 29.

Ameloblastomas consist of odontogenic tissue and abundant tumor stroma including fibroblasts. Our group recently determined that interactions between ameloblastoma tumor cells and stromal fibroblasts are often conveyed by various cytokines including interleukin (IL)‐1α, IL‐6, and IL‐8, which create a microenvironment favorable to tumor invasion 20. Thus, these findings suggest that stromal cells may induce collective cellular invasion in these tumors. As such, the present study examined this event with two immortalized fluorescence‐labeled cell lines derived from plexiform or follicular ameloblastomas in 3D culture, as well as the functional significance of fibroblasts in this process.

Previous studies on collective invasion using 3D culture models have focused on neural crest cell migration or epithelial cancer invasion 15, 30, both of which indicate that mesenchymal cells affect the collective migration of epithelial cells. In this study, we used HFF‐2 fibroblasts that did not stem from ameloblastoma–stroma as an established immortalized fibroblast cell line 31. While ameloblastoma‐associated fibroblasts seem to be preactivated and stimulated the proliferation and invasive activity of head and neck cancer cells 17, HFF‐2 fibroblasts expressed similar levels of cytokine mRNA in response to the stimulation with ameloblastoma‐derived conditioned medium as well as primary ameloblastoma‐associated primary fibroblasts 20. Considering this, we speculated that HHF‐2 fibroblasts could work well for the *in vitro* 3D culture experimental model for collective migration of ameloblastoma cells surrounded by fibroblasts.

Although ameloblastoma cell invasion with 3D culture method was examined 16, collective cellular invasion of ameloblastoma or the participation of stromal fibroblasts has not been specifically assessed. The DL‐CGH system is a superior method to evaluate the collective cellular invasive form of cells 26.

Notably, our study demonstrated that AM‐1 and AM‐3 tumor cells exhibit different collective cellular invasive patterns. In single cultures, plexiform AM‐1 cells displayed sharp processes that invaded into the surrounding collagen, whereas follicular AM‐3 cells showed blunt processes characteristic of collective invasions. Significantly, these results indicated that the different tumor types exhibit distinct characteristics with respect to cellular migration, intercellular adhesion, and cytoskeleton. Moreover, we examined gene expression in AM‐1 and AM‐3 cells using DNA microarray and identified several cytokines that were markedly upregulated in AM‐3 cells as compared to AM‐1 counterparts (data not shown), suggesting that cytokine expression may be responsible for the histological differences. Furthermore, the collective invasion of tumor cells was changed under the presence of fibroblasts. In the presence of fibroblasts, both AM‐1 and AM‐3 tumor cells displayed an increased number of invasive processes, which extended to the outer layer. However, it should be noted that the invasive phenotype was far more pronounced in the AM‐3 cell line.

We performed three patterns of DL‐CGH methods (ameloblastoma cells only, ameloblastoma cells coated with fibroblasts, and mixed coculture of ameloblastoma cells with fibroblasts) and observed the different cellular behaviors. We have shown that the presence and localization of fibroblasts affected the motility of ameloblastoma cells. The cellular behaviors of ameloblastoma cells coated with fibroblasts using DL‐CGH resembled real histological finding that the stroma including fibroblasts and collagen surrounds around tumor parenchyma of ameloblastoma. On the other hand, the coculture model of ameloblastoma and fibroblasts using DL‐CGH was thought to mimic the early stage of the disease, where we could assume that epithelial and mesenchymal cells exist in a mixed condition. Both of DL‐CGH culture models might be useful for the studies about various stages of ameloblastoma. For example, soluble factors, their neutralizing antibodies, agonists, or antagonists can be applied to inner or outer collagen gels of DL‐CGH to analyze their effects toward collective migration/invasion.

In this study, we reported the cellular behaviors (collective migration) of ameloblastoma cells stimulated with or without fibroblasts. Previously, we investigated differences in gene expression on several factors associated with cellular invasion in the past study. For example, the expressions of MMP series such as MMP‐9, which is a digestive protease, are different between AM‐1 and AM‐3 cells 19. In that study, we have shown that Wnt‐3a promotes the expression of MMP‐9 from ameloblastoma cells. MMP series have crucial role in bone destruction, and the difference in expression of these factors could affect the behavior of the tumor, such as invasiveness 27. Other group reported that TNF‐α induced the expression IL‐6 and MMP‐9 from ameloblastoma cells 32. However, we have not yet clarified factors that influence the differences in collective cellular invasion in ameloblastoma. Given that Girdin, an actin‐binding substrate of PI3 kinase, is important for the collective migration of neuroblasts 15, unidentified factors similar to Girdin in function might regulate the collectiveness of the behaviors of ameloblastoma. It is necessary to investigate these factors playing an important role in clinical behaviors such as tumor invasion and recurrence in ameloblastoma.

In conclusion, the present study clarified the characteristic differences in ameloblastoma tumors *in vitro*. In addition, we demonstrated that fibroblasts potentiate collective cellular invasion form of the ameloblastoma tumor cells, which is crucial for understanding of disease state. As such, it is necessary to identify the factors influencing these differences in ameloblastoma subtypes—particularly with respect to tumor–stroma interactions—in order to develop effective therapies to treat this disease in the future.

## Author contributions

TF, MK, MI, TKibe, HK, MU, YM, and TKiyono performed the experiments. YN analyzed the data. NN and SK developed the concept and designed the experiments. TF, HK, and SK wrote the manuscript.
